# Comment on: “35 Years of Joyner’s Endurance Performance Model: Assessing the Contribution of Physiological Determinants of Performance Proxies in 888 Individuals from Recreational to World Class”

**DOI:** 10.1007/s40279-026-02507-3

**Published:** 2026-08-03

**Authors:** Sebastian Keller, Sanghyeon Ji, Patrick Wahl

**Affiliations:** 1 Section Exercise Physiology, German Sport University, Am Sportpark Müngersdorf 6, 50933 Cologne, Germany; 2 The German Research Centre of Elite Sport Cologne, German Sport University, Cologne, Germany

Dear Editor,

We read with great interest the recent original research article by Mougin et al. [1], which uses the classical physiological model of endurance performance to assess the contribution of physiological determinants in running and cycling in a large cohort of 888 athletes of different performance levels and both sexes. The authors conclude that maximal oxygen uptake ($\dot{\mathrm{V}}$O_2_max), followed by exercise economy, explained most of the variance (~ 95%) in speed or power output at lactate thresholds (LTs) as endurance performance proxies, with only a limited contribution of fractional utilization of $\dot{\mathrm{V}}$˙O_2_max at LTs. These are undoubtedly important findings with clear implications for training prescription and athlete profiling, for which the authors are to be congratulated. In an effort to advance the discussion, we would like to raise several points regarding previous related work and methodological considerations.

The research deficit motivating the study was formulated as follows: “Most supporting studies have been limited by small sample sizes (often < 20 participants) and homogeneous male cohorts”. While this may be correct for many earlier studies, it overlooks Ji et al. [2], published in the European Journal of Applied Physiology, which applied the classical physiological model to explain running speed at LT2 in 45 male and 55 female young athletes. A follow-up study by Fischer et al. [3], also published in the European Journal of Applied Physiology, pursued a similar approach in young cycling athletes including 61 males and 22 females. In light of this previous work, the claim that “no large-scale study has yet examined their relative contribution to performance variation for running and cycling in a large cohort of athletes, across sexes and performance levels” appears misleading: a combined total of 100 running [2] and 83 cycling athletes [3] is entirely consistent with what would typically be considered a large-scale study in sports science research [4], and both cited articles explicitly quantified the relative contributions of the physiological model parameters for running and cycling separately by sex.

In terms of the contributions, there is also a methodologically relevant distinction between the analytical approaches employed. Building on multiple linear regression analysis, similar to Mougin et al. [1], Ji et al. [2] and Fischer et al. [3] employed commonality analysis to determine how much variance in the criterion was uniquely explained by each predictor, independent of all other predictors (unique effects), and how much variance was shared by combinations of predictors (common effects). In contrast to the Lindeman, Merenda and Gold method used by Mougin et al. [1], which distributes shared variance among predictors by averaging marginal contributions across all possible predictor orderings, the decomposition of R^2^ through commonality analysis takes into account dependencies between predictors including suppressor effects [5]. This distinction is not merely statistical; in Ji et al. [2], the suppression effect between $\dot{\mathrm{V}}$˙O_2_max (referred to as “$\dot{\mathrm{V}}$˙O_2_peak”) and exercise economy (referred to as oxygen cost of running)—reflecting their interaction in defining maximal aerobic speed—amounted to − 19.5% to − 51.7% of total R^2^, varying systematically between male and female subgroups. This is a substantively important physiological finding that the Lindeman, Merenda and Gold method is structurally unable to detect. Although Mougin et al. [1] verified the absence of collinearity using variance inflation factors, it is well established that $\dot{\mathrm{V}}$˙O_2_max and exercise economy may be correlated [6, 7], making commonality analysis particularly informative in this context.

This is particularly important as the absence of a (statistically significant) correlation between $\dot{\mathrm{V}}$˙O_2_max and exercise economy in a large and heterogeneous group does not preclude possible differences in relationships among subgroups. For example, Fischer et al. [3] demonstrated that the correlation between $\dot{\mathrm{V}}$˙O_2_peak and exercise economy (referred to as oxygen cost of cycling) reverses direction when moving from the heterogeneous overall sample to more homogeneous performance subgroups. The large dataset of Mougin et al. [1], comprising three predefined performance tertiles per sex and modality (which still provide sufficient sample sizes), would have been particularly well suited to exploring this aspect through within-tertile regression (potentially combined with commonality) analyses. This would have meaningfully refined the practical interpretation of the model across the performance continuum. For instance, Mougin et al. [1] mention in their discussion that $\dot{\mathrm{V}}$˙O_2_max is likely already maximized in elite athletes, suggesting that the other two physiological parameters may be more important. This theoretical but plausible assumption could have been directly tested using tertile-specific decomposition analyses to reveal whether exercise economy or fractional utilization becomes more important in higher performing athletes.

In general, a methodological issue concerns the potential mathematical dependency between the model predictors and the criterion when using LT, as in previous studies [2, 3, 8, 9]. This applies particularly to exercise economy when determined at LT, as in Mougin et al. [1]; since this variable is calculated by dividing oxygen uptake by running speed or mechanical power output at LT, the criterion (speed or power at LT) appears implicitly on both sides of the equation, which will artificially inflate model accuracy and the variance explained. Evidence of this inflation is visible in the Bland–Altman plots of Mougin et al. [1], whereby limits of agreement are considerably narrower when modeling speed at LT (approximately − 0.55 to + 0.45 km·h^−1^) than at lactate turnpoint (analogous to LT2) (approximately − 0.88 to + 0.80 km·h^−1^; Fig. 6C and 6D; the same pattern holds for cycling in panels G and H), consistent with a stronger circular dependency at LT. In this context, the R^2^ values of 94–99% reported by Mougin et al. [1] (in particular the R^2^ values for LT ≥ 98%) should be interpreted as reflecting internal model consistency rather than genuine predictive validity, and the characterization of “very high predictive accuracy” warrants qualification. In contrast, previous studies from our and other groups using external criteria yielded R^2^ values that, while still high, tended to be lower; for example, for performance in a 16-km running time trial (R^2^ = 95% [10]), in a 15-km cycling time trial (R^2^ = 85% [11]), and for speed or power at maximal lactate steady state in running and cycling (R^2^ = 83% and 79%, respectively [12]). Therefore, when modeling LT—especially when this is included in the calculation of exercise economy—circularity is an important limitation that warrants explicit acknowledgement, as mentioned by Fischer et al. [3].

Besides LT modeling in a large sample, we acknowledge that Mougin et al. [1] address a broader set of questions, including LT ratios and heart rate as practical intensity proxies. The evaluation of these aspects, as well as the contribution of physiological parameters to the classical physiological model that has been used for even more than 35 years to predict endurance performance (e.g., [13, 14]), is considered valuable for assessing endurance performance and deriving individual athlete profiles. Nonetheless, we respectfully submit the aforementioned points to advance the discussion, ensure that the existing literature is accurately reflected, and encourage the adoption of analytical approaches that most comprehensively reveal the physiological structure underlying endurance performance.
